# Assessment of Changes in Concentration of Total Antioxidant Status, Acute-Phase Protein, and Prolactin in Patients with Osteoarthritis Subjected to a Complex Spa Treatment with Radon Water: Preliminary Results

**DOI:** 10.1155/2020/9459418

**Published:** 2020-04-23

**Authors:** Jadwiga Kuciel-Lewandowska, Michał Kasperczak, Lilla Pawlik-Sobecka, Małgorzata Paprocka-Borowicz, Jan Gnus

**Affiliations:** ^1^Department of Physiotherapy Medical, University of Wroclaw, Wrocław, Poland; ^2^Department of Clinical Nursing Medical, University of Wroclaw, Wrocław, Poland

## Abstract

Spa treatment brings many clinical benefits such as improved physical activity, pain relief, and improved quality of life. In the literature, there are only few objective studies evaluating changes in metabolism possibly influencing clinical outcomes. The main purpose of our study was the assessment of the effect of spa treatment on changes in concentration of TAS, CRP, and PRL in patients with osteoarthritis. Patients receiving spa treatment were enrolled. TAS, CRP, and PRL levels were obtained using standard tests before the beginning of treatment as well as on days 5 and 18. The study group consisted of *n* = 35 patients with peripheral joint and spinal osteoarthritis. The control group consisted of 15 people selected from the resort staff, who also suffered from osteoarthritis and had no contact with radon. An increase in TAS concentration was found in the study group following therapy while the control group was characterized by a significant decrease in TAS. On day 5, an increase in TAS concentration was found in both groups, however, with much worse result in the control group. No changes in CRP concentration were statistically significant. PRL concentration was proven to decrease in a statistically significant way after treatment in the study group. This trial is registered with NCT03274128.

## 1. Introduction

Spa treatment brings many positive clinical benefits such as improved physical activity, pain relief, and improved quality of life. In the literature, there are only few objective studies evaluating changes in metabolism possibly influencing clinical outcomes. Due to much controversy regarding spa treatment, we have attempted to prove the effect of such a therapy on selected metabolic parameters. In our study, TAS, CRP, and PRL were considered.

In living organisms oxygen reacting with organic compounds leads to their oxidation accompanied by partial reduction of oxygen itself due to various external and internal factors, and as a consequence reactive, oxygen species (ROS) are produced. ROS include singlet oxygen ^1^O_2_, superoxide O^−^_2_, hydrogen peroxide H_2_O_2_, and hydroxyl radical OH, and they are responsible for intercellular damage with subsequent cell apoptosis. When in full health, the balance between removal and creation of ROS is strictly controlled and maintained. Increased ROS production or decrease in their removal leads to oxidative stress [1–3]. The clinical sequelae of this include cellular damage and development of numerous disorders such as atherosclerosis, Alzheimer's disease, Parkinson's disease, inflammation, arthritis, allergies, cancer, diabetes, and macular degeneration [4–6]. The human body can be protected from ROS effects by different metabolic pathways both regulating and blocking free radical production.

Antioxidant defence mechanisms include

1. Endogenous antioxidants produced by the organism such as (a) antioxidant enzymes (superoxide dismutase, glutathione peroxidase, and catalase); (b) nonenzymatic antioxidants (linolenic acid, polyamides, albumin, bilirubin, glutathione, uric acid, ceruloplasmin, transferrin, and Q10 coenzyme), which all have different targets
2. Exogenous antioxidants provided from the environment such as vitamins C, A, E, carotenoids, xanthophylls, and polyphenols

Those compounds directly interact with free radicals and influence cellular signal transduction, activity of enzymes and genes participating in cellular apoptosis, and DNA repair [7, 8]. Evaluation of the antioxidant system can be done in several ways, including total antioxidant status or its components separately, especially those from the first group. In this study, the total antioxidant concentration in plasma, or TAS, was assessed. The integrated antioxidant system was evaluated, including all biological components involved in antioxidation activity.

### 1.1. CRP

The acute-phase protein, or C-reactive protein (CRP), is a heterogeneous class of proteins synthesized in the liver. The CRP concentration raises in acute and chronic inflammation. The biological properties of CRP include complement activation, initiation of phagocytosis, and protection against autoimmunization. CRP accumulates in damaged tissues, inducing complement activation and attracting macrophages. CRP can also transport materials released after tissue necrosis. Due to those properties, CRP is not only an inflammation marker, but also actively takes part in it, as the main function of CRP is bounding and detoxification of certain biological particles. It is assumed that from all acute-phase proteins, only CRP fulfills, to a limited degree, diagnostic requirements and can be used in the following instances: for inflammation detection, prognosis of disease development, and monitoring of response to treatment. The usefulness of this marker in the diagnosis and prevention of cardiovascular diseases, type 2 diabetes, and Crohn's disease has been shown in the recent years. According to Koj, the use of CRP as a marker is still a subject of studies and different diagnostic applications [9, 10].

### 1.2. Prolactin

Prolactin (PRL) is a peptide hormone synthetized and secreted mainly by lactotropic cells of the posterior lobe of the pituitary gland. It is mainly produced in the form of prehormone with molecular mass equal to 26 kDa, which later undergoes partial proteolysis and forms final polypeptide with a molecular mass of 23 kDa. The three main isoforms of PRL include the little or free PRL, big PRL with molecular mass 50-60 kDa, and big PRL (BB-PRL) with molecular mass 150-204 kDa. All those isoforms are reactive in diagnostic tests. The main function of prolactin is activating growth hormone and development of mammary glands as well as synthesis and secretion of milk. It also participates in the neurobiological adaptation of the body to pregnancy and lactation, which leads to characteristic changes in behaviour and formation of maternal instincts. In men, PRL influences the morphology and function of testicles as well as epididymis, seminal glands, prostate, and sperm. It is one of the hormones participating in stress reaction. It also demonstrates analgesic properties. Moreover, it takes part in regulating fluid-electrolyte balance, reduces water and sodium loss in kidneys (due to increased sodium reabsorption), promotes sodium reuptake in intestines, and reduces sodium and chloride loss with sweat. Prolactin is also an immunomodulatory factor affecting immune response. It also takes part in the pathogenesis of autoimmune diseases [11, 12].

Radon (or radon-active) water refers to a characteristic type of water containing trace amounts of radon, an unstable radioactive element, as well as products of its decay. Radon water can be used in therapy if the radon content exceeds 74 Bq/l (2nCi/l), and if it meets both extraction and hygienic requirements. Radon is a chemical element resulting from radioactive decay of uranium and thorium. It is classified as a noble gas. It is colourless and odourless, and it dissolves well in water, especially when water is poorly mineralized or acidic. Radon has many isotopes among which radon-222 is the precursor of other isotopes, formed as a direct product of alpha decay of radium-226. The emitted alpha particles have small penetration depth but high ionizing potential. The half-life of radon is 3.8 days. Radon concentration in natural conditions is subject to constant daily changes as well as seasonal changes caused by precipitation. Radon loss can be observed during therapy due to technical causes such as accumulation in water reservoirs, transfer within pipes, heating, cooling, and intensive exploitation (radon concentration drops between 40% and 80% have been reported). Such a variation of radon concentration at the source makes dose calculation impossible and impractical, so it is not routinely applied. Radon is in 95% absorbed and in 90% excreted through the lungs, the rest being excreted through kidneys or skin. During baths, the absorption is mainly through airways, because radon and its derivatives accumulate above the water surface in high concentration. Lungs are especially exposed to radon because the decay products form deposits in the alveoli. Radioactive sludge makes durable deposits on the skin which can persist for a couple of hours. Radioactive decay within the body varies greatly depending on the fat content. Also the adrenal cortex, liver, and muscles play an important role in this process. Anti-inflammatory, desensitizing, and analgesic activity of radon can be explained by activating the adrenal cortex and increased production of steroid hormones. Studies showed increased levels of luteinizing hormone, growth hormone, cortisol, testosterone, estradiol, and estriol in plasma. Radon-based therapies improve peripheral circulation, reduce oedema, reduce joint and muscular pain, as well as increase the general level of fitness. The following have also been observed: decreased blood pressure, reduced cholesterol and triglyceride levels, decreased erythrocyte sedimentation rate, increased haemoglobin and erythrocytes count, increased level of ionized calcium, increased parathormone and calcitonin levels, and accelerated removal of harmful metabolic by-products [13–16]. Low-dose ionizing radiation is used in spa therapy in Świeradów-Zdrój resort. Complex spa therapy includes different treatment modalities such as kinesiotherapy, physical treatment with healing lights, electrotherapy, ultrasound therapy, cryotherapy, peak magnetic field at low and high frequencies, hydrotherapy with different waters (salty, hydrogencarbonate, and radon), and peloid therapy—*borowina* (turf) wraps and baths. *Borowina* is a peat formed in water as a result of decomposition under a lack of oxygen. It contains many minerals, hormones, proteins, fats, tannins, and so-called humic acids. Series of spa balneophysiotherapy treatment causes nonspecific changes in the body's adaptive reactivity. Spa medicine also includes prevention, health education, and psychological counselling.

The purpose of our study was the assessment of the effect of spa therapy, consisting of peloid therapy, physical therapy, kinesiotherapy, and radon water, on changes in concentration of TAS, CRP, and PRL in patients with osteoarthritis.

## 2. Materials and Methods

The study was conducted in Świeradów-Zdrój health resort. Blood samples of 1 mL were obtained using closed-system venepuncture from the ulnar vein before initiation of treatment as well as on day 5 and 18 of treatment. Blood samples were then centrifuged and the blood plasma was kept at +6°C, and standard laboratory tests were later performed.

The study group consisted of *n* = 35 patients. The age of the patients ranged from 47 to 63 years. The mean age was 56.5 years. The study group consisted of 24 females and 11 males. Patients with peripheral joint and spinal osteoarthritis were enrolled in the study. The major inclusion criteria were peripheral joint or spinal osteoarthritis, age between 45 and 65 years, informed consent, and no contradictions for complex spa therapy. Exclusion criteria were as follows: age below 45 years and above 60 years and contraindications to spa therapy. Patients received basic or light diet, mostly consisting of low-fat meals. Both diets had normal calorie intake. Vitamin supplements were not used.

The control group consisted of 15 people aged 50 to 62 years with a mean age of 54.2 years, selected from the resort staff, among which 9 were women and 6 were men. The control group included individuals with osteoarthritis, who did not receive spa treatment (i.e., no contact with radon). The control group had the same inclusion and exclusion criteria as well as the same examination procedures as the study group.

Therapeutic water was assumed as the main therapeutic factor. Świeradów-Zdrój is characterized by low-mineralized water, and the main therapeutic factor is its radon activity—Rn 303.1-441.5 Bq/l. Differences in radon concentrations are characteristic for different spas and are considered their unique features.

The complex spa therapy consisted of therapeutic radon water, peloid therapy with *borowina*, cryotherapy, massage, physical therapy, and kinesiotherapy. Radon treatment was characterized by the following parameters: full radon baths—temperature 37°C, duration 15 min., and every other day; radon inhalations through mouth—duration 15 min., temperature 37°C, and every other day. Baths and inhalations were performed alternately—the total number of radon treatment sessions was 15. Patients were recommended to take 10 sessions of different therapeutic modalities, depending on their physical condition and medical history. Dosage and duration of treatment were chosen according to current standards. The sample treatment plan was radon baths and inhalations, peloid wraps (*borowina*), therapeutic exercises (both in group and individually), biostimulation with lasers, and interferential currents.

Other treatment modalities and their doses are as follows:

- *borowina* treatment (peloid therapy)—partial peloid wraps, duration 20 min, temperature 40-42°C
- healing exercises in swimming pool with normal water
- individual exercises with machines and group exercises chosen individually for each patient, taking into account his or her fitness level; mean duration was 30-45 minutes
- field therapy—walks, exercises in the fresh air
- dry massage—depending on the specific patient's needs involving cervical spine (CC), thoracic spine (TH), or lumbar spine (LS);
- laser therapy—parameters: sweep, continuous work, wavelength 808 nm, power 12.0 J, 400 mV, duration 30 s
- low-frequency magnetic field: duration 20 min, rectangular signal, induction 5 mT, frequency 20-50 Hz
- ultrasound therapy—parameters: head 800 kHz/6 cm^2^, impulse ultrasound wave 2 ms impulse, 9 ms break, dose 0.5-0.6 W/cm^2^, duration 6 minutes
- cryotherapy—airflow, duration 2-3 minutes, temperature between -80°C and -110°C
- electrotherapy: diadynamic currents (Bernard's currents)—parameters: DF1 CP4 LP4, Nemec interferential currents (frequency ranged between 0 Hz and 100 Hz), percutaneous electrical stimulation (TENS)—impulse rectangular current, duration of impulse 0.2 ms, frequency 40 Hz, and electric current regulated between 0 mA and 100 mA
- light therapy—Sollux lamp with blue filter, distance 30-40 cm, duration 15 minutes, Bioptron lamp—distance 10 cm, duration 5-10 min

Spa therapy model, including radon therapy, has more than a hundred-year tradition in Poland and Europe. Therapeutic approaches were modified according to actively developing hydrotherapy and balneology. Radon therapy in Poland may be prescribed only by a physician after considering possible indications and contraindications, and it is based on an established standard dosage. The number and type of treatment sessions, including inhalations, baths, or mouthwash, are determined beforehand. Radon spas use water from natural sources extracted from drills performed in comply with the mining law, after obtaining permission by the Ministry of Environment and under the supervision of the Ministry of Health. Every therapeutic water must comply with balneochemical and bacteriological criteria to be recognized as therapeutic water.

Therapeutic water and rooms in radon spas are subject to constant dosimetry monitoring. It allows to quantify patient's exposure to radiation. Calculation of the absorbed dose is not performed because it varies depending on patient's body built, in particular on fat content and absorption surface (of the respiratory tract), comorbidities, as well as extraction loss. Dosimetric measurements are conducted every day using certified detectors.

Our study was conducted as a part of the Polish Radon Cluster. The Polish Radon Cluster initiative was established on 12 November 2014, and it unites radon spas in Poland, which offer large-scale radon medical treatment. Partners of the Polish Radon Cluster include the Medical University of Wrocław, Local Activity Forum Association, and Sudetian Business Incubator, Świeradów-Czerniawa Spa ltd.—PGU group, Lądek-Długopole Spa SA. Polish Radon Cluster mission is to make use of the therapeutic and rehabilitation potential of spa corporations, which manage mining areas abundant in radon waters, as voluntary cooperation of entrepreneurs, natural and legal persons, and research units. The goal of this initiative is to create a modern, national radon therapeutic centre in Poland, particularly in Lower Silesia region based on research results and know-how of the Polish Radon Cluster participants.

We obtained the permission of the Bioethical Commission of Medical University of Wrocław—opinion No 135/2015, signed consent of the spa president, and individually signed informed consent of each participant, according to standards recommended by the Bioethical Commission.

For the statistical analysis of TAS results, we used the Statistica 12 software. The mean values (measures of location), standard deviation (measure of spread), and range (extreme values) were calculated for numeric variables. Each quantitative variable was examined with the Shapiro-Wilk test to assess the distribution type. A comparison of results between measurements in each group was conducted using Friedman's ANOVA test and post hoc test. For all comparisons, we assumed that *α* = 0.05 and *p* values were rounded up to four decimal places. For CRP and PRL, the statistical analysis was conducted using the Statistica 13 software (StatSoft, Inc., USA). For numeric variables, arithmetic means, median, standard deviation, quartiles, and range (extreme values) were calculated. Each quantitative variable was examined using the Shapiro-Wilk test to assess the distribution type. A comparison of results between the study group and the control group was conducted using Mann-Whitney *U* test. Comparison of subgroup results between tests I, II, and III was conducted using nonparametric Friedman's rank ANOVA test. For all comparisons, the significance level *α* = 0.05 was assumed and *p* values were rounded up to four decimal places.

## 3. Results

**Figure 1:**
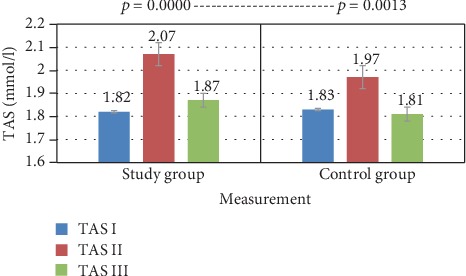
TAS—comparison of means in both groups for three tests—Friedman's ANOVA analysis of variance.

In the study group, an increase in the TAS level was observed in tests II and III. In the control group in test II, there was an increase in the TAS level, while in test III the TAS decreased below the baseline. The result analysis showed statistically significant changes in the study group and slightly worse results in the control group. Multiple comparisons of Friedman's ANOVA post hoc test when comparing tests I and II, as well as II and III showed statistically significant changes in the study group (increase in TAS concentration). As for the control group, the increase in TAS concentration in test II was statistically significant. However, a comparison of measurements II and III showed a decrease in TAS concentration and is statistically significant. When comparing tests I and III for both groups, the *p* value does not meet the assumed significance level (Table 1 and Figure 1).

**Figure 2:**
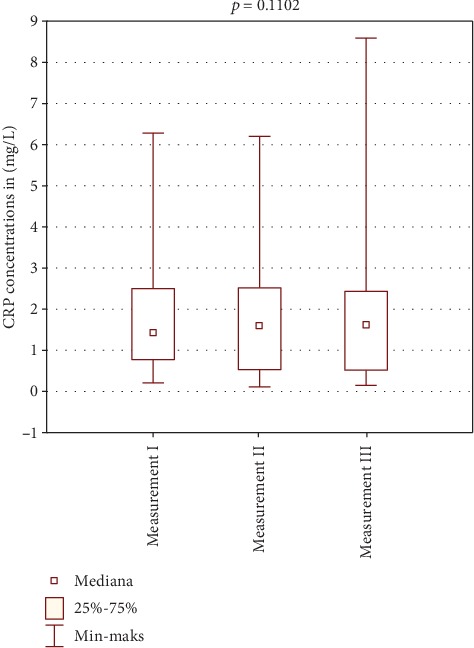
Intragroup comparison of CRP results between tests I, II, and III—study group.

**Figure 3:**
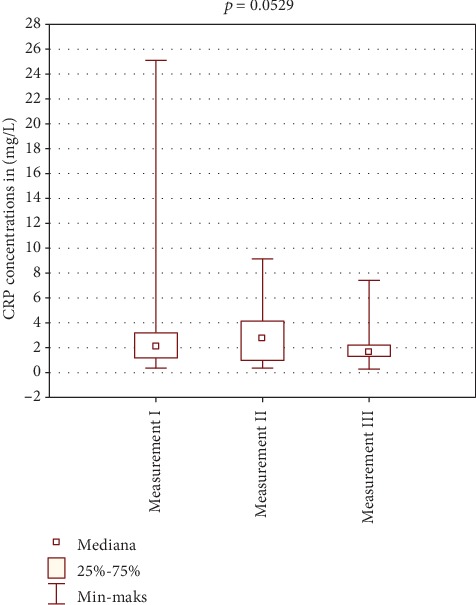
Intragroup comparison of CRP results between tests I, II, and III—control group.

**Figure 4:**
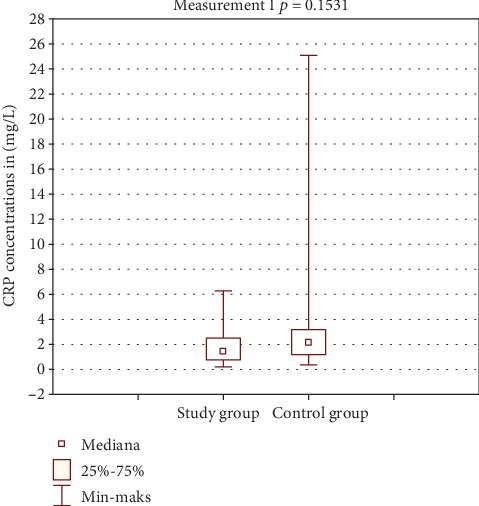
Comparison of CRP results between groups—test I.

**Figure 5:**
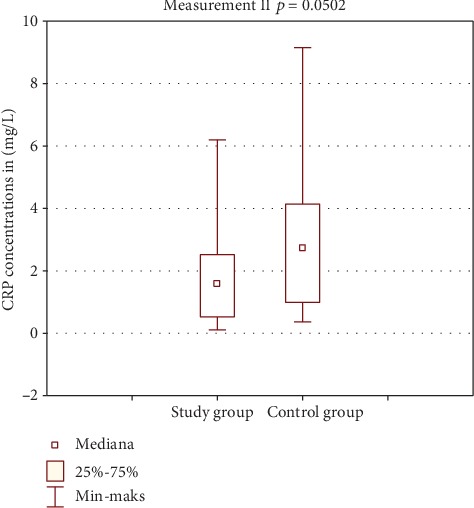
Comparison of CRP results between groups—test II.

**Figure 6:**
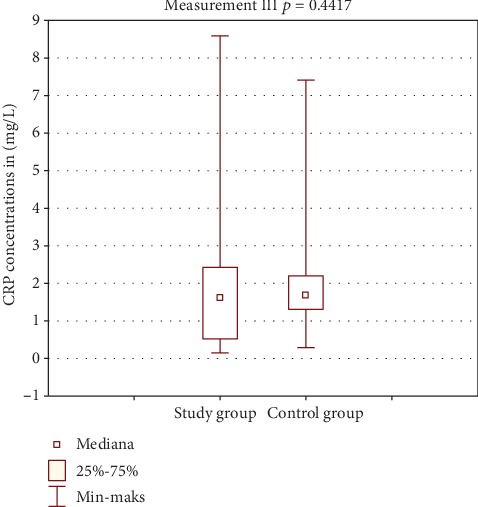
Comparison of CRP results between groups—test III.

Table 2 shows the results of CRP concentration in the study and control group. Figures 2 and 3 show comparison of intragroup results between the first, second, and third test. Figures 4, 5, and 6 show result comparison between the study and control group.

**Figure 7:**
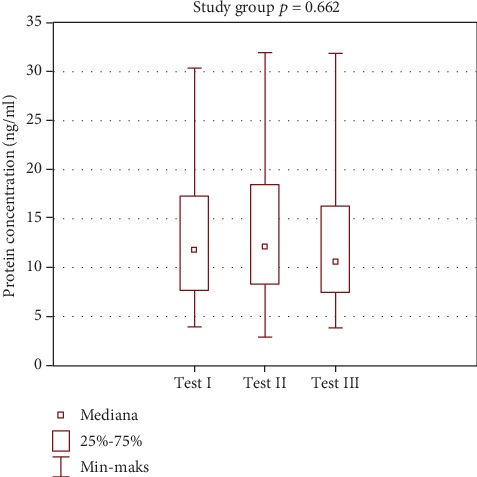
Intragroup comparison of prolactin level—study group.

**Figure 8:**
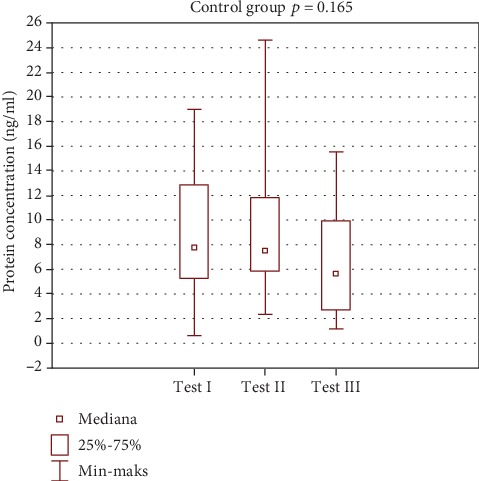
Intragroup comparison of prolactin level—control group.

Globally, greater changes in prolactin level were observed in the study group. However, those were not statistically significant. Test III showed a statistically significant difference between the study and control group. A statistically significant decrease in prolactin concentration was observed in the study group. Such a result may suggest the impact of spa balneotherapy on fluctuations in this hormone level. Low magnitude of changes in the control group may support this hypothesis. Changes in prolactin levels are shown in Table 3 and Figures 7 and 8.

Intragroup comparison (Friedman's rank ANOVA).

After treatment, the TAS level increased in the study group while TAS decreased significantly in the control group. On day 5, an increase in TAS was observed in both groups with a significantly worse result in the control group. No statistically significant changes in concentration were observed for CRP. Measurements of PRL concentration in the study group showed a statistically significant decrease following treatment.

## 4. Discussion

In the study group, an increase in TAS concentration following treatment was observed, and in the control group, a decrease in the total antioxidative status was found, which may indicate the impact of the spa therapy. In both groups, an increase in total antioxidative status was found in test II. In the study group, there was a statistically significant change in TAS concentration with *p* = 0.0001, while in the control group *p* = 0.0071 (Table 1). The obtained TAS results may indicate the sensitivity of the antioxidant system to the effects of spa therapy. The increase in TAS concentration in the study group was due to the activation of the body's antioxidant system in response to the applied therapy. In the control group, however, the increase in TAS on day 5 is unclear and the change in value is less significant. In addition, the final result indicates a decrease in TAS concentration in this group, which could ultimately indicate the absence of an antioxidant stimulating factor.

Activation of the antioxidation system may affect a number of metabolic pathways that have been discussed in the introduction. It is currently difficult to assess to what extent spa therapy and ionizing radiation stimulate this system. There are few publications to be referred to when discussing our results. Most articles published in the 1960s to 1980s and animal studies conducted in Japan only discussed the effects of radon therapy in very general terms. Mainly analgesic effect, swelling reduction, and improved physical performance were reported. Later, studies have shown that radon reduces the release of free oxygen species in patients with ankylosing spondylitis disease, has an antiproliferative effect on cancer cells, and stimulates enzyme activity including peroxide dismutase [17–21]. Most publications discuss the health effects of exposure to ionizing radiation but only with respect to specific exposure. There are studies of populations living in areas with naturally high radiation or populations living in areas of previous atomic explosion or occupational exposure. Those studies have shown that low doses of radiation have a neutral or positive effect on health [22–24].

In the study group, the CRP concentration from three tests was within the normal limits. Comparison of mean and median CRP concentrations in tests I, II, and III showed statistically insignificant changes *p* = 0.1102 (Figure 2). In the control group, the CRP concentration changes in the three tests were also within the normal limits. Statistical analysis showed changes in CRP concentration in this group on the edge of statistical significance *p* = 0.0529 (Figure 3). Those fluctuations in CRP levels were within normal limits and were not of clinical relevance. Comparison of results between the study and control group in tests I and III was not statistically significant (Figures 4 and 6). Test II showed a statistically significant difference with *p* = 0.0502 (Figure 5).

The lack of significant changes in CRP measurements was also confirmed in the study by Franke et al. [16]. Olah et al. obtained different results. They showed statistically significant and persistent (three months after a balneotherapy course) decrease in serum C-reactive protein concentration in patients treated with therapeutic thermal baths [25]. Misztela et al. showed a statistically insignificant decrease in CRP concentration as a result of artificial sulfur baths in patients receiving spa treatment. Most importantly, clinical improvement was observed [26]. Ponikowska et al. showed the effect of mud products, among others, on the CRP level [27].

In the study group, there were more pronounced changes in prolactin levels. The results were not statistically significant. Test III showed a statistically significant difference between the study and control groups. A statistically significant decrease in prolactin concentration was observed in the study group. Such results may suggest an effect of spa balneotherapy on fluctuations of prolactin level. Less pronounced changes in the control group may support this hypothesis (Table 3, Figures 7 and 8).

It should be emphasized that spa treatment, regardless of whether it is directed towards hormonal disorders or not, has a significant impact on the endocrine gland activity. The influence of spa treatment on the secretion of “stress hormones,” manifested by a significant increase in the secretion of cortisol and ACTH, as well as an increase in the secretion of growth hormone and prolactin, has been observed, while no effect of spa treatment on the circadian rhythm of secretion of any of the above-listed hormones has been observed [28, 29]. A significant increase in prolactin level was observed after hyperthermal procedures. Studies have shown that the increase in prolactin secretion, caused by overheating in the sauna, was similar to that seen under stress or during exercise [30]. In the context of the prolactin role in patients with osteoarthritis, a decrease rather than an increase in the level of this hormone, of course within the reference range, seems beneficial. It should prompt to avoid treatments that cause an increase in prolactin concentration.

To sum up, in the case of spa medicine, obtaining positive effects of therapy depends on the type and intensity of stimuli used, body's reactivity, individual sensitivity, presence of concomitant disorders, and genetic conditioning of enzymatic pathways. The use of natural healing raw materials, e.g., healing waters, is particularly difficult in therapeutic practice due to their low pharmacodynamic stability. Due to the metabolic activity of chemical compounds contained in medicinal waters, the fundamental therapeutic principle should be individual selection and individualization of therapy as well as further research in this field. In the case of radioactive water, the absorbed radiation dose, the extent of systemic changes, and loss of radiation intensity during the distribution of radioactive water are challenging. The final result of spa treatment is always the sum of the activities of each form of therapy. The assessment of the positive impact of spa therapy on the human body requires conducting multidisciplinary research. It is necessary to design randomized studies conducted on large groups of patients.

## 5. Conclusions

The influence of spa treatment with radon water on the total antioxidant status has been proven, together with a decrease in prolactin concentration among patients treated at the resort. The influence of spa treatment on the acute-phase protein (CRP) concentration has not been confirmed.

## Figures and Tables

**Table 1:** TAS—comparison of both groups.

	TAS mmol/l	*N*	$\overset{®}{x}$	Min	Max	SD	*p* ^∗^	*p* ^∗∗^
Study group	Measurement I	35	1.82	1.55	2.06	0.14	0.0000	I vs. II—*p* = 0.0001 I vs. III—*p* = 0.3788 II vs. III—*p* = 0.0001
	Measurement II	35	2.07	1.80	2.53	0.20		
	Measurement III	35	1.87	1.71	2.13	0.12		

Control group	Measurement I	15	1.83	1.65	2.09	0.11	0.0013	I vs. II—*p* = 0.0071 I vs. III—*p* = 0.9444 II vs. III—*p* = 0.0030
	Measurement II	15	1.97	1.81	2.21	0.12		
	Measurement III	15	1.81	1.62	2.10	0.14		

*N*: number of trials; $\overset{®}{x}$: mean; Min: minimum; Max: maximum; SD: standard deviation; ^∗^analysis of variance ANOVA Friedman; ^∗∗^multiple comparisons in post hoc ANOVA Friedman test.

**Table 2:** CRP concentration (mg/l)—statistical analysis—descriptive statistics—study and control group.

Variable	Group	*N*	$\overset{®}{x}$	Me	Min	Max	Q1	Q3	SD
Test I	Study	35	1.91	1.43	0.21	6.28	0.77	2.50	1.59
Test II	Study	35	1.73	1.60	0.11	6.20	0.53	2.52	1.43
Test III	Study	35	1.78	1.61	0.15	8.59	0.52	2.43	1.68
Test I	Control	15	4.06	2.12	0.37	25.09	1.18	3.19	6.28
Test II	Control	15	2.98	2.74	0.37	9.15	0.99	4.14	2.47
Test III	Control	15	2.08	1.67	0.29	7.41	1.31	2.20	1.67

$\overset{®}{x}$: mean; Me: median; Min: minimum value; Max: maximum value; Q1: lower quartile; Q3: upper quartile; SD: standard deviation.

**Table 3:** Comparison of prolactin level between groups.

	Study group (*n* = 35)							Control group (*n* = 15)							*p* ^∗^
Variable	$\overset{®}{x}$	Me	Min	Max	Q1	Q3	SD	$\overset{®}{x}$	Me	Min	Max	Q1	Q3	SD	
Test I	13.6	11.7	3.9	30.4	7.6	17.3	7.6	8.8	7.7	0.6	19	5.2	12.8	5.2	0.056
Test II	13.1	12.1	2.9	32	8.3	18.4	6.6	9.6	7.5	2.3	24.6	5.8	11.8	6.5	0.081
Test III	12.9	10.6	3.8	31.9	7.4	16.3	7.3	6.7	5.6	1.2	15.6	2.7	9.9	4.6	0.004
Difference II/I	-0.6	-0.9	-12.6	13.4	-2.9	1.8	5.3	0.8	0.5	-7.2	10.8	-1.3	1.8	5.1	0.596
Difference III/I	-0.7	-0.1	-10.3	4.8	-3.8	2.4	3.9	-2.1	-2.1	-15.9	4.2	-3.4	1.6	5	0.478
Difference III/II	-0.2	0.6	-12.4	13.7	-2.7	2.3	5.1	-2.9	-1.9	-18.5	6.3	-4.4	2.4	7.1	0.318

$\overset{®}{x}$: mean; Me: median; Min: minimum value; Max: maximum value; Q1: lower quartile; Q3: upper quartile; SD: standard deviation; ^∗^Mann-Whitney *U* test.

## Data Availability

All data are contained and described within the manuscript. The datasets used and/or analyzed during the current study available from the corresponding author on reasonable request.
